# A Secure and Efficient Authentication Scheme with Privacy Protection for Internet of Medical Things

**DOI:** 10.3390/s26010313

**Published:** 2026-01-03

**Authors:** Feihong Xu, Jianbo Wu, Qing An, Rahman Ziaur

**Affiliations:** 1 Hubei Engineering Research Center for BDS-Cloud High-Precision Deformation Monitoring, School of Artificial Intelligence, Wuchang University of Technology, Wuhan 430223, China; 120160287@wut.edu.cn; 2 Department of Information and Communication Technology, Mawlana Bhashani Science and Technology University, Tangail 1902, Bangladesh; zia@mbstu.ac.bd

**Keywords:** Internet of Medical Things, biomedical sensor, data security, privacy-preserving, authentication

## Abstract

The Internet of Medical Things represents a pivotal application of Internet of Things technology in Healthcare 4.0, offering substantial practical benefits in enhancing medical quality, reducing costs, and minimizing errors. In history, researchers have proposed numerous privacy-preserving authentication schemes to safeguard Internet of Medical Things applications. Nevertheless, due to design shortcomings, existing solutions still encounter significant security and performance challenges, rendering them impractical for real-world use. To resolve the issue, this work introduces a novel practical Internet of Medical Things-based smart healthcare system, leveraging a pairing-free certificateless signature scheme and hash-based message authentication code. Through formal security proofs under standard cryptographic assumptions, and performance analysis, our scheme demonstrates enhanced security while maintaining desirable computational and communication efficiency.

## 1. Introduction

The Internet of Things (IoT) builds a dynamic interactive intelligent network platform by seamlessly connecting numerous intelligent sensing devices, embedded systems, and the Internet. It not only realizes real-time data collection, transmission, and collaborative processing between devices, but also promotes revolutionary application scenarios in smart home, industrial automation, smart city, and medical health. The Internet of Medical Things (IoMT) emerges as a critical application subset within this framework, where healthcare devices/applications are intricately integrated with healthcare IT systems to furnish patients with high-quality health services. According to recent market insights from Grand View Research, the global IoMT market is anticipated to exceed USD 658.57 billion by 2030 [1], propelled by innovations in wearable technology, remote patient monitoring, and AI-driven diagnostics. This significant growth trajectory highlights the transformative impact of IoMT on healthcare in the Healthcare 4.0 era, promoting proactive disease management and the implementation of tailored treatment strategies [2].

In typical IoMT application environments, such as wireless body area networks and wireless medical sensor networks, wearable or implanted sensors on the human body capture vital health metrics, including blood oxygen saturation, temperature, heart rate, and respiratory rate [3,4]. Due to the constrained resources of these sensor devices, the gathered data are transmitted to a remote medical cloud server (MCS) for storage and subsequent analysis. Subsequently, healthcare providers can retrieve the patient’s health information (PHI) from the MCS to deliver prompt medical interventions.

Normally, PHI is frequently transmitted across inherently insecure public networks, such as the internet and wireless channels, where it faces a spectrum of security risks that compromise both its integrity and confidentiality. For example, during transmission, PHI may suffer from inadvertent or malicious tampering, leading to altered medical records or distorted diagnostic images. Such modifications can mislead healthcare providers into making clinically erroneous decisions. Beyond integrity threats, PHI inherently contains highly sensitive personal data. Unauthorized disclosure of this information, whether through eavesdropping, data breaches, or insufficient access controls, carries severe privacy repercussions [5,6]. For example, the harm from health data leakage may include financial fraud, identity theft, and reputational damage, leading to discrimination or stigma, and can also affect physical and mental well-being. It is said that healthcare breaches are the costliest of any industry, with average costs per incident reaching approximately USD 7.42 million in 2025. Breaches specifically involving IoMT average even higher, at an estimated USD 10 million per attack [7]. Therefore, these integrity and confidentiality risks underscore the critical need for robust privacy-preserving authentication frameworks to protect PHI.

Throughout the history of IoMT security research, numerous privacy-preserving authentication schemes have been proposed to ensure data integrity and authenticity [8]. Early implementations primarily rely on two kinds of cryptographic mechanisms, namely, public key infrastructure (PKI) and identity-based cryptography (IBC) [9,10]. However, both mechanisms exhibit inherent limitations in resource-constrained IoT applications. In particular, PKI-based systems demand substantial overhead for key certificate management, such as certificate issuance, distribution, update, and revocation, which are expensive for medical sensors. Meanwhile, an IBC system can address the certificate management problem. However, it suffers from the key escrow problem, where a key generation center (KGC) knows both the private and public keys of the user. In [9], Kumar et al. designed an escrow-free identity-based aggregated signcryption scheme for IoMT. They used an interactive system key generation method to address the key escrow problem. However, their design lacks formal security analysis, and the heavyweight bilinear pairing and map-to-point hash operations are unfriendly to resource-constrained medical sensors.

To eliminate the key management and key-escrow problems, a pivotal advancement emerged when the concept of certificateless cryptography (CLC) was introduced in [11]. The CLC paradigm redefines key generation by splitting user keys into two components, namely, a partial secret derived from a KGC and a user-generated private value. By eliminating both certificate management burdens and key escrow vulnerabilities, CLC strikes an optimal balance between security and efficiency. This also makes CLC an ideal cryptographic system for deployment in resource-limited IoMT applications.

### 1.1. Related Work and Motivation

Prior to that, to protect data integrity and confidentiality at the same time, many CLC-based privacy-preserving authentication schemes have been constructed for IoMT environments. Liu et al. [12] designed an RSA-based certificateless signcryption (CLSC) scheme for healthcare applications. Such a scheme can merge the digital signature and encryption operations into a single logical step. However, their computational and communication costs pose a performance challenge for resource-constrained sensor devices. Subsequently, some new CLSC schemes were proposed one after another, such as [13,14,15,16]. Considering the significant increase in the number of sensors in IoMT, the traditional mode of verifying signcrypted messages one by one can easily lead to network congestion. To this end, researchers have proposed a number of certificateless aggregate signcryption (CLASC) schemes that support batch signcryption verification, which have become a critical path for optimizing communication efficiency in IoMT scenarios [15,16,17,18,19,20,21,22]. However, these solutions still have some shortcomings in terms of security and performance.

In addition to signcryption construction, in [23], Chang et al. introduced a new IoMT-based smart healthcare system (SHS) on the basis of a homomorphic certificateless signature (CLS) scheme and a hash-based message authentication code (HMAC) [24]. Compared to the conventional CLS scheme, their homomorphic CLS scheme can achieve public verifiability, i.e., allowing internal or external entities to check the integrity of data without knowing the actual PHI data stored in MCS [9]. Meanwhile, the HMAC construction can encrypt the PHI by using only XOR and hash operations, which are quite lightweight. However, in [25], Xu et al. demonstrated that the design in [23] cannot resist signature forgery attacks by public key replacement attackers. In particular, such an attack allows the attacker to extract the signer’s full private key, thereby compromising the basic data integrity guarantee. To address the issue, Xu et al. proposed a security-enhanced CLS scheme and designed a new IoMT-based SHS. However, using theoretical analysis methods, their design still has security vulnerabilities. More concretely, in their design, each biomedical sensor encrypts its encrypted data to a personal-assisted device (PAD), which then signs the data and further sends the data–signature pair to an MCS for subsequent processing. In this process, the PAD does not know the actual PHI data. However, in real-world environments, it cannot detect whether this encrypted data has been unintentionally or maliciously tampered with during transmission. Note that once the data integrity is compromised, the patient may receive incorrect medical services, causing significant health risks. In summary, Table 1 compares the features of several related works. Therefore, there still exists a research gap for an IoMT-based SHS that provides both data integrity and confidentiality assurance.

In view of this, this work aims to answer the following research question: Can we design a practical IoMT-based SHS that can simultaneously protect data integrity and confidentiality?

### 1.2. Contribution

To answer the above research question, we mainly design a practical IoMT-based SHS. The scheme overview is shown in Section 3. The main contributions are as follows:

- We design a new IoMT-based SHS based on a new pairing-free CLS signature and the ChaCha20-Poly1305 algorithm. Our solution achieves data integrity and privacy protection throughout the entire process from data generation to data usage.
- We formally prove the security of our design based on standard cryptographic assumptions in the random oracle (RO) model.
- Through comparative evaluation with existing research, we assess the efficacy of our proposed scheme. The results show that our solution has ideal computational and communication costs while ensuring high security, making it suitable for resource-constrained IoMT applications.

### 1.3. Road Map

The structure of the remaining paper is as follows: We revisit required preliminaries in Section 2. Section 3 presents our new IoMT-based SHS with related security proofs. Section 4 evaluates its performance, and Section 5 ends the paper.

## 2. Preliminaries

We introduce some preliminaries in this section, including general symbols, the elliptic curve discrete logarithm problem (ECDLP), and the ChaCha20-Poly1305 algorithm.

### 2.1. Symbols

Some symbols are described in Table 2.

### 2.2. ECDLP

Let *G* be a *q*-order cyclic elliptic curve group and *P* be a generator of *G*. Given αP∈G for some unknown $\alpha \in Z_{q}^{*}$, the ECDLP’s goal is to find α.

### 2.3. ChaCha20-Poly1305

ChaCha20-Poly1305 [26] is an authenticated encryption algorithm that combines the ChaCha20 stream cipher for confidentiality and the Poly1305 message authentication code for integrity and authenticity. It is a fast, secure, and efficient symmetric-key algorithm used in protocols like Transport Layer Security and Secure Shell to protect data. At a high level, ChaCha20-Poly1305 consists of three algorithms:

- CP.KeyGen: Given a security parameter ζ, the algorithm returns a 32-byte key $k_{cp}$.
- CP.Enc-Auth: Given a message $m\in {\left\{0,1\right\}}^{*}$, a 12-byte random nonce iv, an a variable length associate data $t\in {\left\{0,1\right\}}^{*}$, and the key $k_{cp}$, the algorithm returns a ciphertext *c* and a 16-byte tag τ.
- CP.Verify: Given the key $k_{cp}$, ciphertext *c*, tag τ, nonce iv, and associate data *t*, the algorithm returns a message *m* or ⊥ indicating decryption failure.

We refer the readers to [26] for a detailed description of the ChaCha20-Poly1305.

## 3. The Proposed IoMT-Based SHS

**Figure 1:**
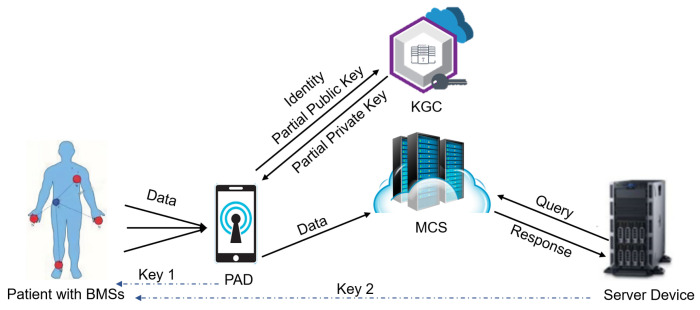
Structure of the system.

To illustrate the IoMT-based SHS, similar to prior work [9,23,25], we consider one patient as a concrete example. As depicted in Figure 1, five core entities interact within the system: The KGC mainly builds the system and issues the partial private key to the PAD. Wearable/implantable biomedical sensors (BMSs), with constrained resources, continuously collect PHI. During System Setup, each BMS securely obtains two ChaCha20-Poly1305 keys from the PAD and SD, respectively, which it subsequently uses for PHI encryption and authentication. This process can be achieved through authenticated key-exchange protocols [27], which is beyond the scope of the work. Acting as a data aggregator, the PA receives encrypted PHI from multiple BMSs, checks the data integrity, signs the “compressed” ciphertext, and transmits the validated data to a MCS. On the healthcare provider side, the SD authorizedly access stored PHI through MCS to deliver healthcare services to the patient.

In the following, we will introduce the detailed implementation of our proposed SHS, which integrates a new CLS scheme with a secure ChaCha20-Poly1305 mechanism (using algorithms (CP.KeyGen, CP.Enc-Auth, CP.Verify)), whose specifications are given in Section 2.3.

### 3.1. System Setup

The KGC sets up the system by generating the required system parameters. PAD and SD interact with KGC, respectively, to establish their public–private key pairs. Without loss of generality, we assume that a PAD is linked to *n* BMSs. Algorithm 1 provides a detailed description of the phase.
**Algorithm 1** System Setup.1:Given a security parameter ζ, the KGC sets a system master key $\alpha \in Z_{q}^{*}$ at random and public parameters $ppa=\{G,P,q,PK_{kgc},H_{i}\}$. Specifically, G is a *q*-order cyclic group, *P* is a generator of G, and $PK_{kgc}=\alpha P$. In addition, $H_{i}:{\left\{0,1\right\}}^{*}\to Z_{q}^{*}$, i=1,2,…,3 are cryptographic hash functions.2:To generate a public–private key pair, the PAD with identity $ID_{i}$ picks a secret value $x_{i}\in Z_{q}^{*}$ at random and calculates $X_{i}=x_{i}P$. It provides KGC with $\left(ID_{i},X_{i}\right)$. Then, the KGC picks $r_{i}\in Z_{q}^{*}$ at random, calculates $R_{i}=r_{i}P$, $h_{1i}=H_{1}\left(ID_{i},X_{i},R_{i},P_{kgc}\right)$, $d_{i}=r_{i}+\alpha h_{1i}$, and sends $D_{i}=\left(d_{i},R_{i}\right)$ to the PAD as its partial private key. After checking the correctness of $d_{i}$ by verifying $d_{i}P=R_{i}+h_{1i}P_{kgc}$, the PAD sets its private key $SK_{i}=\left(x_{i},d_{i}\right)$ and public key $PK_{i}=\left(X_{i},R_{i}\right)$.3:
For *j*-th BMS, the PAD executes CP.KeyGen(ζ) to generate a symmetric private key $k_{cp1j}$. The SD also executes CP.KeyGen(ζ) to generate the symmetric private key $k_{cp2j}$. Then, $\left(k_{cp1j},k_{cp2j}\right)$ are securely stored in the non-volatile memory of the *j*-th BMS.

### 3.2. Data Flow from BMS to PAD

This part describes how the BMS sends its collected data to the PAD. Assuming that $m_{j}$ is the PHI gathered by BMS_*j*_, 1≤j≤n, at time *t*, Algorithm 2 provides a detailed description of the phase.
**Algorithm 2** BMS-to-PAD data sharing.1:
For $m_{j}\in {\left\{0,1\right\}}^{\zeta }$, BMS_*j*_ randomly picks 12-byte $iv_{1j},iv_{2j}\in {\left\{0,1\right\}}^{*}$, calculates $\left(c_{1j},\tau_{1j}\right)=\mathrm{CP}.\mathrm{Enc}-\mathrm{Auth}\left(k_{cp2j},m_{j},iv_{2j},t\right)$, where (associate data) *t* is a timestamp. For $M_{j}=\left(c_{1j},\tau_{1j}\right)$, BMS_*j*_ further computes $\left(c_{1j}^{\prime },\tau_{1j}^{\prime }\right)=\mathrm{CP}.\mathrm{Enc}-\mathrm{Auth}\left(k_{cp1j},M_{j},iv_{1j},t\right)$ and sets $M_{j}^{\prime }=\left(c_{1j}^{\prime },\tau_{1j}^{\prime }\right)$.2:
Send $\left\{ID_{j},t,M_{j}^{\prime },iv_{1j},iv_{2j}\right\}$ to PAD for further processing.

### 3.3. Data Flow from PAD to MCS

For each received item $\left\{ID_{j},t,M_{j}^{\prime },iv_{1j},iv_{2j}\right\}$ with regard to BMS_*j*_, the PAD first checks and confirms its validity. Then, it generates a signature σ for messages sent by *n* BMSs, and sends the message–signature pair to the MCS for subsequent processing. Note that in this phase, the PAD does not access the actual PHI as the transmitted message component $M_{j}$ is securely maintained in encrypted format. Algorithm 3 shows the phase.
**Algorithm 3** PAD-to-MCS data sharing.1:
Check and decrypt $M_{j}=\mathrm{CP}.\mathrm{Verify}\left(k_{cp1j},M_{j}^{\prime },iv_{1j},t\right)$ for 1≤j≤n.2:For $M_{1},M_{2},\ldots ,M_{n}$, the PAD calculates $M=H_{2}\left(M_{1},M_{2},\ldots ,M_{n}\right)$. It randomly picks $u_{i}\in Z_{q}^{*}$ and calculates $U_{i}=u_{i}P$ and $h_{3i}=H_{3}\left(M,ID_{i},PK_{i},U_{i},t\right)$. Then, it computes $V_{i}=u_{i}+h_{3i}\left(x_{i}+d_{i}\right)$ and sets $\sigma_{i}=\left(U_{i},V_{i}\right)$ as the signature.3:Send the item $\left\{ID_{i},{\left\{ID_{j},M_{j},iv_{2j}\right\}}_{j=1}^{n},t,\sigma_{i}\right\}$ to MCS for storage.

### 3.4. Data Access

To facilitate patient-specific medical services utilizing data aggregated by the MCS, the SD must securely access authenticated and encrypted PHI data $\left\{ID_{i},{\left\{ID_{j},M_{j},iv_{2j}\right\}}_{j=1}^{n},t,\sigma_{i}\right\}$. The operational workflow for this phase is defined in Algorithm 4.
**Algorithm 4** Data access.1:The SD downloads the data $\left\{ID_{i},{\left\{ID_{j},M_{j},iv_{2j}\right\}}_{j=1}^{n},t,\sigma_{i}\right\}$ from the MCS.2:Compute $M=H_{2}\left(M_{1},M_{2},\ldots ,M_{n}\right)$, $h_{i2}=H_{2}\left(ID_{i},X_{i},R_{i},P_{kgc}\right)$, and $h_{3i}=H_{3}\left(M,ID_{i},PK_{i},U_{i},t\right)$.3:Use the equation $V_{i}P=U_{i}+h_{3i}\left(X_{i}+R_{i}+h_{1i}P_{kgc}\right)$ to check the validity of $\sigma_{i}$. The correctness:$$\begin{array}{cc} V_{i}P & =(u_{i}+h_{3i}\left(x_{i}+d_{i}\right))P=u_{i}P+h_{3i}\left(x_{i}+d_{i}\right))P\\ & =U_{i}+h_{3i}\left(x_{i}P+d_{i}P\right)=U_{i}+h_{3i}\left(X_{i}+R_{i}+h_{1i}P_{kgc}\right). \end{array}$$The SD stops the verification if $\sigma_{i}$ is invalid; otherwise, it operates as follows.4:Check and decrypt $m_{j}=\mathrm{CP}.\mathrm{Verify}\left(k_{cp2j},M_{j},iv_{2j},t\right)$ for 1≤j≤n.

### 3.5. Security Proof

Now, we prove the security of our IoMT-based SHS. Note that our design consists of an underlying CLS scheme and the ChaCha20-Poly1305 algorithm. For the CLS scheme, two types of adversaries should be considered, namely, a public-key replacement attacker (called a Type 1 adversary) and a malicious-but-passive KGC (called a Type 2 adversary). In particular, a Type 1 attacker knows a target user’s secret value. However, the adversary cannot access the user’s partial private key. In addition, a Type 2 attacker knows the KGC’s private key but cannot know the target user’s secret value. For more security definitions and security models, we refer the readers to [28] for details.

**Theorem 1.**  *In the RO model, if the underlying CLS scheme and ChaCha20-Poly1305 algorithm are secure, then our IoMT-based SHS is secure.*

**Proof.**  This theorem demonstrates that if an attacker exists who can compromise the security of our SHS, then another attacker must exist who can break either the underlying CLS scheme or the ChaCha20-Poly1305 algorithm. As ChaCha20-Poly1305 algorithm is specified in RFC 7539, we omit its security analysis. Given this in mind, the proof for our theorem incorporates the demonstrations detailed in Theorems 2–4. □

**Theorem 2.**  *Our underlying CLS scheme is secure against a Type 1 adversary if the ECDLP is hard.*

**Proof.**  This theorem demonstrates that if a Type 1 adversary $A_{1}$ compromises the underlying CLS scheme, then it must be possible to construct an adversary B that can solve the ECDLP. Now, $A_{1}$ and B perform the following:

- Step-1: B runs as System Setup to obtain system parameters $ppa=\{G,P,q,PK_{kgc},H_{i}\}$, where $P_{kgc}=\alpha P$ for some unknown $\alpha \in Z_{q}^{*}$. It then sends ppa to $A_{1}$. For simplicity, let $ID_{i}^{*}$ be $A_{1}$’s target identity. During the forgery game, $A_{1}$ keeps a series of lists as defined below to record the query results. In the initial stage, these lists are empty.
- Step-2: In this stage, B responds to $A_{1}$’s adaptive queries as below.
  $H_{1}$-Query: When an $H_{1}$ query is received from $A_{1}$ for $\left(ID_{i},X_{i},R_{i},P_{kgc}\right)$, if the item $\left(ID_{i},X_{i},R_{i},P_{kgc},h_{1i}\right)$ exists in the list $L_{H_{1}}$, B returns $h_{1i}$ to $A_{1}$. Otherwise, B picks $h_{1i}\in Z_{q}^{*}$ at random, inserts $\left(ID_{i},X_{i},R_{i},P_{kgc},h_{1i}\right)$ to $L_{H_{1}}$, and responds $h_{1i}$ to $A_{1}$.
  $H_{3}$-Query: For an $H_{3}$ query on $\left(m_{i},ID_{i},PK_{i},U_{i},t_{i}\right)$, if the item $\left(m_{i},ID_{i},PK_{i},U_{i},t_{i},h_{3i}\right)$ exists in the list $L_{H_{3}}$, B returns $h_{3i}$ to $A_{1}$. Otherwise, B randomly picks $h_{3i}\in Z_{q}^{*}$, inserts $\left(m_{i},ID_{i},PK_{i},U_{i},t_{i},h_{3i}\right)$ to the list, and responds $h_{3i}$ to $A_{1}$.
  Secret value-Query: $A_{1}$ can issue such query on $ID_{i}$. B searches the tuple $\left(ID_{i},x_{i},X_{i}\right)$ from the list $L_{sv}$ and provides it to $A_{1}$. Otherwise, B selects $x_{i}\in Z_{q}^{*}$ at random, stores $\left(ID_{i},x_{i},X_{i}\right)$ to $L_{sv}$, and responds $x_{i}$ to $A_{1}$.
  Partial private key-Query: $A_{1}$ can issue such query regarding $ID_{i}$. If $ID_{i}=ID_{i}^{*}$, B reports failure. Otherwise, B finds the tuple $\left(ID_{i},d_{i},R_{i}\right)$ from the list $L_{ppk}$ and then responds it to $A_{1}$. Note that if $\left(ID_{i},d_{i},R_{i}\right)$ does not exist in $L_{ppk}$ and the tuple $\left(ID_{i},X_{i},R_{i},P_{kgc},h_{1i}\right)$ does not exist in $L_{H_{1}}$, B selects $d_{i},h_{1i}\in Z_{q}^{*}$ at random, computes $R_{i}=d_{i}P-h_{1i}P_{kgc}$, and sets $h_{1i}=H_{2}\left(ID_{i},X_{i},R_{i},P_{kgc}\right)$. A updates lists $L_{H_{1}}$ and $L_{ppk}$ and returns $\left(ID_{i},d_{i},R_{i}\right)$ to $A_{1}$.
  Public key-Query: Once B receives $A_{1}$’s query on $ID_{i}$ ($ID_{i}=ID_{i}^{*}$), B checks if $\left(ID_{i},x_{i},X_{i},d_{i},R_{i}\right)$ exists in the list $L_{key}$. If it exists, B returns $\left(X_{i},R_{i}\right)$. Otherwise, B runs as Secret value-Query and Partial private key-Query to generate and update $\left(ID_{i},x_{i},X_{i},d_{i},R_{i}\right)$, and then returns $\left(X_{i},R_{i}\right)$.
  Public key replacement-Query: Once B receives a query for the tuple $\left(ID_{i},PK_{i},PK_{i}^{\prime }\right)$ from $A_{1}$, B searches the tuple $\left(ID_{i},PK_{i}\right)$ from the list $L_{key}$ and replaces it with $\left(ID_{i},⊥,X_{i}^{\prime },d_{i},R_{i}\right)$.
  Signing-Query: For $A_{1}$’s query on $\left(m_{i},ID_{i}\right)$, B performs as below. If $ID_{i}\neq ID_{i}^{*}$, B scans the lists to obtain the required parameters and runs as Signing to generate a signature $\sigma_{i}=\left(U_{i},V_{i}\right)$ as the response. Otherwise, B picks $h_{1i}$, $h_{3i},V_{i}\in Z_{q}^{*}$ at random, sets $U_{i}=V_{i}P-h_{3i}\left(X_{i}+R_{i}+h_{1i}P_{kgc}\right)$, and returns $\sigma_{i}=\left(U_{i},V_{i}\right)$.
- Step-3: Eventually, $F_{1}$ either admits failure or returns its forgery $\sigma_{i}^{*}=\left(U_{i}^{*},V_{i}^{*}\right)$ on $m_{i}^{*}$.

If in the case that $\sigma_{i}^{*}$ is a valid forgery under $\left(ID_{i}^{*},m_{i}^{*}\right)$, the verification equation $V_{i}^{*}P=U_{i}^{*}+h_{3i}^{*}\left(X_{i}^{*}+R_{i}+h_{1i}^{*}P_{kgc}\right)$ holds. By applying the forking lemma in [29], B replays $A_{1}$ with the same random tape, but provides two distinct values of $H_{1}$ hash. $A_{1}$ can output another valid signature $\sigma_{i}^{*}=\left(U_{i}^{*},V_{i}^{{*}^{\prime }}\right)$. Hence, we have $V_{i}^{{*}^{\prime }}P=U_{i}^{*}+h_{3i}^{*}\left(X_{i}^{*}+R_{i}+h_{1i}^{{*}^{\prime }}P_{kgc}\right)$. Therefore, B calculates $\alpha =\left(V_{i}^{*}-{V_{i}^{*}}^{\prime }\right){\left(h_{3i}^{*}\left(h_{1i}^{*}-h_{1i}^{{*}^{\prime }}\right)\right)}^{-1}$ as a solution to the ECDLP. □

**Theorem 3.**  *Our underlying CLS scheme is secure against any Type 2 adversary if the ECDLP is hard.*

**Proof.**  This theorem demonstrates that if a Type 2 adversary $A_{2}$ compromises the underlying CLS scheme, then it must be possible to construct an adversary B that can solve the ECDLP. Now, $A_{2}$ and B perform the following:

- Step-1: B runs as System Setup to obtain system parameters $ppa=\{G,P,q,PK_{kgc},H_{i}\}$, where $P_{kgc}=\alpha P$ and $\alpha \in Z_{q}^{*}$. It then sends (ppa,α) to $A_{2}$. For simplicity, let $ID_{i}^{*}$ be $A_{2}$’s target identity. During the forgery game, $A_{2}$ keeps a series of lists as defined below to record the query results. In the initial stage, all lists are empty.
- Step-2: In this phase, B responds to $A_{2}$’s adaptive queries. The queries $H_{1}$-Query, $H_{3}$-Query, Public key replacement-Query, and Signing-Query are the same as in the proof of Theorem 2.
  Secret value-Query: $A_{2}$ can issue the secret value query on $ID_{i}$. If $ID_{i}=ID_{i}^{*}$, B aborts. Otherwise, B searches the tuple $\left(ID_{i},x_{i},X_{i}\right)$ from the list $L_{sv}$ and returns it to $A_{2}$. Otherwise, B selects $x_{i}\in Z_{q}^{*}$ at random, stores $\left(ID_{i},x_{i},X_{i}\right)$ to $L_{sv}$, and responds $x_{i}$ to $A_{2}$.
  Partial private key-Query: For $A_{2}$’s query on $ID_{i}$, B operates as the following: If $ID_{i}=ID_{i}^{*}$, B aborts. Otherwise, B checks $L_{sv}$ to find $\left(ID_{i},x_{i},X_{i}\right)$, picks $h_{1i},r_{i}\in Z_{q}^{*}$ at random, computes $R_{i}=r_{i}P$, and sets $d_{i}=r_{i}+\alpha h_{1i}$ and $h_{1i}=H_{1}\left(ID_{i},X_{i},R_{i},P_{kgc}\right)$. Then, it inserts $\left(ID_{i},x_{i},X_{i},d_{i},R_{i}\right)$ and $\left(ID_{i},X_{i},R_{i},P_{kgc},h_{1i}\right)$ to lists $L_{key}$ and $L_{H_{1}}$, respectively, and returns $D_{i}=\left(d_{i},R_{i}\right)$ to $A_{2}$.
  Public key-Query: Once B receives $A_{1}$’s query on $ID_{i}$, B performs the steps as below. If $ID_{i}\neq ID_{i}^{*}$, B runs as Secret value-Query and Partial private key-Query to obtain and update $\left(ID_{i},x_{i},X_{i},d_{i},R_{i}\right)$, and then returns $\left(X_{i},R_{i}\right)$. Otherwise, B first sets $x_{i}=⊥,X_{i}=\beta P$ for some unknown $\beta \in Z_{q}^{*}$, and operates as Partial private key-Query to generate $D_{i}=\left(d_{i},R_{i}\right)$. Then, B records the item $\left(ID_{i},⊥,X_{i},d_{i},R_{i}\right)$ to $L_{key}$ and returns $\left(X_{i},R_{i}\right)$.
- Step-3: Eventually, $F_{1}$ either admits failure or returns its forgery $\sigma_{i}^{*}=\left(U_{i}^{*},V_{i}^{*}\right)$ on $m_{i}^{*}$.

If in the case that $\sigma_{i}^{*}$ is a valid forgery under $\left(ID_{i}^{*},m_{i}^{*}\right)$, the verification equation $V_{i}^{*}P=U_{i}^{*}+h_{3i}^{*}\left(X_{i}^{*}+R_{i}+h_{1i}^{*}P_{kgc}\right)$ holds. By applying the forking lemma in [29], B replays $A_{2}$ with the same random tape, but provides two distinct values of $H_{3}$. $A_{1}$ can output another valid signature $\sigma_{i}^{*}=\left(U_{i}^{*},V_{i}^{{*}^{\prime }}\right)$. Hence, we have $V_{i}^{{*}^{\prime }}P=U_{i}^{*}+h_{3i}^{{*}^{\prime }}\left(X_{i}^{*}+R_{i}+h_{1i}^{*}P_{kgc}\right)$. Therefore, B obtains two independent equations satisfying $V_{i}^{*}=U_{i}^{*}+h_{3i}^{*}\left(\beta +d_{i}\right)$ and $V_{i}^{{*}^{\prime }}=U_{i}^{*}+h_{3i}^{{*}^{\prime }}\left(\beta +d_{i}\right)$ [30]. Therefore, B can compute the value of β, which is a solution to the ECDLP. □

**Theorem 4.**  *Our SHS achieves privacy preservation if the underlying ChaCha20-Poly1305 algorithm is secure.*

**Proof.**  To protect the privacy of PHI data, in our design, each BMS adopts a widely used ChaCha20-Poly1305 algorithm to encrypt data. As fully analyzed by Bellare et al. in [31], this encrypt-then-MAC paradigm provides both privacy and integrity. In particular, the privacy property inherently implies the security of indistinguishability under chosen-ciphertext attacks. We omit the rigorous proof here for simplicity. □

## 4. Performance Evaluation

This section presents a comparative performance analysis of our proposed SHS, benchmarking its computational and communication costs against prior art solutions detailed in studies [9,19,23,25].

The proposed SHS system consists of four sequential phases: System Setup (A), BMS-to-PAD data sharing (B), PAD-to-MCS data sharing (C), and SD’s data access (D). As stage A can be executed once during the offline phase to complete the establishment of system parameters and entity registration, we will omit the performance comparison for this stage.

### 4.1. Computational Costs Comparison

To evaluate the computational efficiency of these schemes, we set up a benchmark experiment for testing several cryptographic operations. More concretely, a Raspberry Pi 3B+ device simulates the BMS, while a PC equipped with a 2.5 GHz Intel Core i5-13400 processor and 16 GB RAM simulates the PAD and SD. In particular, to test related cryptographic operations, the secp256k1 curve defined by $E:y^{2}=x^{3}+ax+b\;\;\mathrm{mod}\;q$ is used for schemes based on the elliptic curve cryptography (ECC), where the prime *q* is 32 bytes and $a,b\in Z_{q}^{*}$. To achieve the same security level (i.e., about 128 bit), for schemes, we leverage the bilinear pairing $e:G_{1}\times G_{1}\to G_{T}$, $G_{1}$ is a $\bar{q}$-order group constructed on a BLS12-381 curve $\hat{E}:y^{2}=x^{3}+4\;\mathrm{mod}\;\bar{p}$, where primes $\bar{p}$ and $\bar{q}$ are 48 bytes and 32 bytes, respectively.

For ease of presentation, the time cost for one pairing operation, modular exponentiation operation, pairing-related point multiplication operation, pairing-related point addition operation, ECC-related point multiplication operation, ECC-related point addition operation, map-to-point hash, and a general hash are denoted by the symbols bp, e.bp, pm.bp, pa.bp, pm.ec, pa.ec, mtp, and *h*, respectively. We also use cp.enc and cp.ver to denote encryption and decryption operations related to Chacha20-Poly1305 algorithm, respectively. Specifically, omitting the lightweight XOR operation, the running times for these different operations are presented in Table 3.

In phase B of our design, BMS_*j*_ needs to compute two encryption operations to obtain $M_{j}^{\prime }$. Hence, the time cost for this phase is 2cp.enc=15.092 ms. In the next phase, the PAD executes *n* encryption operations and one general hash operation to check the validity of $M_{j}^{\prime }$ and obtain *M*. Then, to generate a signature σ on *M*, it computes one ECC-related point multiplication and one general hash operation. Hence, the total cost of this phase is pm.ec+2h+2cp.enc=0.691n+1.853 ms. In phase D, the SD executes one general hash to check *M*. Then, it computes two general hash, three ECC-related point multiplication, and three point addition operations to verify σ. After that, it computes *n* decryption operations to obtain $m_{j}$. Therefore, the total cost of this phase is 3pm.ec+3pa.ec+3h+2cp.enc=0.691n+5.688 ms. Similarly, we calculate the computational costs of the schemes [9,19,23,25], and the results are provided in Table 4.

**Figure 2:**
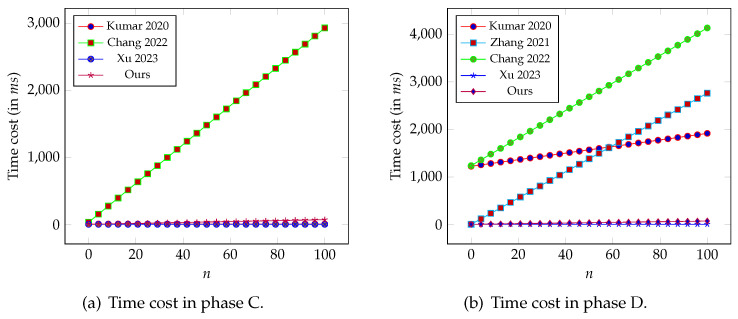
Comparison of computational cost [9,19,23,25].

As presented in the table, in phase B, compared to the schemes in [9,19], the computational cost of all other schemes, including ours, is the same and quite lightweight. For example, compared with the scheme in [9], our computational efficiency is 465 times higher. To better analyze the cost in phases C and D, we visually present the numerical results in Figure 2. As can be seen from Table 4 and Figure 2, in phase C, the computational overheads of schemes [9,25] are both constant, namely, 6.871 ms and 1.854 ms. The cost is positively correlated with the number of BMS *n* in both [23] and our design. Nevertheless, our solution still achieves a very low computational cost. For example, consider a patient utilizing 50 BMSs (we believe that this quantity is sufficient): the execution times of our proposal and the scheme in [23] are 0.118 ms and 36.403 ms, respectively. At this point, even compared to the most efficient scheme in [25], the gap between our two solutions is acceptable. Also note that, compared to the scheme in [25], our solution can achieve higher security. In phase D, the computational cost of all schemes increases linearly with the size of *n*. As can be seen from Table 4, the computational cost of our solution is lower than that of solutions [9,19,23], but higher than that of solution [25]. For example, when n=50, the time cost of our solution is 40.238 ms, while such a cost for remaining schemes are 1572.61 ms, 1385.551 ms, 2688.274 ms, and 7.59 ms. The above analysis indicates that our proposal achieves ideal computational cost while ensuring enhanced security.

### 4.2. Communication Costs Comparison

We will quantitatively evaluate the communication overhead of our SHS against four prior works in [9,19,23,25] regarding phases B, C, and D. According to the curve parameters mentioned earlier, elements in $Z_{\bar{q}}^{*}$, $G_{1}$, $Z_{q}^{*}$, and G occupy 48 bytes, 32 bytes, 32 bytes, and 64 bytes, respectively [25]. Cryptographic operations employ SHA-256 as the foundational hash function, producing 32-byte digests. We assume that the message is 20 bytes. Auxiliary fields include 4-byte timestamps and 4-byte identity markers [32]. We exclude the cost of the message as it is the same in all schemes. In phase B of our design, the BMS_*j*_ sends $\left\{ID_{j},t,M_{j}^{\prime },iv_{1j},iv_{2j}\right\}$ to the PAD, where $M_{j}^{\prime }=\left(c_{1j}^{\prime },\tau_{1j}^{\prime }\right)$. In this process, $ID_{bms}$ is the identity and *t* is the timestamp. Both $iv_{1j}$ and $iv_{2j}$ are 12-byte nonce. $c_{1j}^{\prime }$ and $\tau_{1j}^{\prime }$ are the outputs of the ChaCha20-Poly1305 algorithm. Therefore, the cost is 4+4+20+12+12+16= 68 bytes.

In the next phase, the PAD sends the tuple $\left\{ID_{i},{\left\{ID_{j},M_{j},iv_{2j}\right\}}_{j=1}^{n},t,\sigma_{i}\right\}$ to the MCS, where $ID_{i}$ is PAD’s identity and the signature $\sigma =(U_{i},V_{i})$. In this regard, the cost is 4+(4+20+12)n+4+32+64=(36n+104) bytes.

**Figure 3:**
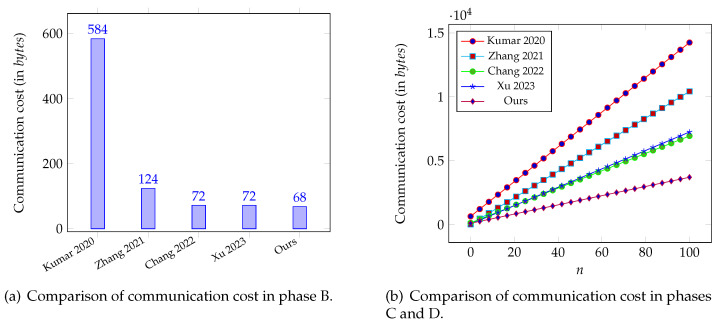
Communication cost comparison [9,19,23,25].

Next, in phase D, the SD downloads the tuple $\left\{ID_{i},{\left\{ID_{j},M_{j},iv_{2j}\right\}}_{j=1}^{n},t,\sigma_{i}\right\}$ from the MCS to obtain the encrypted PHI data for further processing. Hence, the communication cost is quantified as (36n+104) bytes. For comprehensive evaluation, we further count the designs in [9,19,23,25] and present the numerical results in Figure 3.

As presented in Figure 3a, the cost of our design in phase B is lower than the schemes in [9,19,23,25]. In the meantime, Figure 3b reveals that the communication expense of each scheme in phases C and D exhibits a linear growth relative to the number of messages transmitted. Among the compared schemes, our proposed scheme is the most efficient in terms of communication cost scalability in phases C and D. For example, when n=50, the cost for our scheme is 3468 bytes, while the costs of other schemes [9,19,23,25] are 7444 bytes, 5220 bytes, 3532 bytes, 3644 bytes, and 1904 bytes, respectively. In particular, compared to these recent works, the percentage range of our performance improvement is from $\frac{3532-1904}{3532}\approx 46.1\%$ to $\frac{7444-1904}{7444}\approx 74.4\%$.

In summary, it is evident that our proposed system not only has ideal computational efficiency but also maintains the lowest overheads. Consequently, it is well suited for IoMT-based SHS.

## 5. Conclusions

In this paper, we explored the security and privacy issue of IoMT applications. To address the shortcomings in security and performance of existing work, we proposed a new IoMT-based SHS based on a new pairing-free CLS signature and the ChaCha20-Poly1305 algorithm. Our solution can achieve data integrity and privacy protection throughout the entire process from PHI data generation to data usage. We proved the security of our design based on standard cryptographic assumptions in the RO model. We evaluated the performance of our solution by comparing it with relevant work. The results show that our solution has ideal computational and communication costs while ensuring high security, making it suitable for resource-constrained IoMT applications.

Similar to many existing approaches, the private keys of entities in our scheme are used throughout the entire system lifecycle. This limitation lies in the absence of countermeasures for key compromise scenarios. Therefore, building an efficient key update mechanism to achieve forward security is still an open research question. In addition, designing an IoMT-based SHS that is more resource-efficient for sensors with severe resource constraints is also one of our future research interests.

## Figures and Tables

**Table 1:** Feature comparison of our design with related work.

Scheme	F1	F2	F3	F4	F5	F6	F7
[9]	√	√	√	−	−	√	×
[10]	×	×	√	−	×	√	×
[17]	√	√	√	√	√	√	×
[18]	√	√	×	×	√	×	√
[20]	√	√	×	×	√	√	×
[16]	√	√	×	×	√	×	×
[23]	√	√	×	×	√	×	×
[25]	√	√	×	√	√	√	√
Ours	√	√	√	√	√	√	√

F: feature; F1: address key management problem; F2: address key-escrow problem; F3: achieve data integrity; F4: resist public key replacement attack; F5: resist malicious-but-passive KGC attack; F6: achieve data confidentiality; F7: without expensive pairing/exponentiation operation. √ or ×: A property is achieved/exists or not; −: not mentioned.

**Table 2:** Symbols.

Symbols	Descriptions
ζ	System security parameter
$\left(\alpha ,PK_{kgc}\right)$	Master private/public key of the system
ppa	Public parameters of the system
$ID_{i}$	Identity of entity *i*, i∈{BMS,PAD}
$k_{cp1}/k_{cp2}$	Key of ChaCha20-Poly1305
$k_{i}$	A random authentication key
$\left({SK_{ID}}_{i},{PK_{ID}}_{i}\right)$	PAD’s full private–public key pair
*t*	Timestamp
$c_{j}$	Ciphertext for $m_{j}$
$\tau_{j}/\tau_{j}^{\prime }$	ChaCha20-Poly1305-related tag for $m_{j}$
σ	Signature on *M*

**Table 3:** Cryptographic operations with their time costs (in ms).

Symbols	bp	e.bp	pm.bp	pa.bp	pm.ec	pa.ec	mtp	*h*	cp.enc	cp.ver
Time (BMS side)	6790.140	107.585	120.192	0.340	24.948	0.630	56.986	0.013	7.546	7.533
Time (PAD/SD side)	408.935	22.082	6.870	0.023	1.851	0.044	3.499	0.001	0.720	0.691

**Table 4:** Computational cost comparison (in ms).

Scheme	B	C	D
[9]	bp+pm.bp+2mtp+3h ≈7024.343	h+pm.bp ≈6.871	3bp+npm.bp+2(n−1)pa.bp+(n+1)h ≈6.917n+1226.76
[19]	3pm.bp+2pa.bp+3h ≈361.295	−	(4n+1)pm.bp+(4n−3)pa.bp+3nh ≈27.575n+6.801
[23]	*h* ≈0.013	(n+1)e.bp+(n+1)pm.bp+mtp ≈28.952n+32.451	ne.bp+(n+1)pm.bp+3bp+2mtp+h ≈28.952n+1240.674
[25]	*h* ≈0.013	pm.ec+3h ≈1.854	4pm.ec+3pa.ec+(n+4)h ≈0.001n+7.54
Ours	2cp.enc ≈15.092	pm.ec+2h+n cp.ver ≈0.691n+1.853	3pm.ec+3pa.ec+3h+n cp.ver ≈0.691n+5.688

## Data Availability

The data underlying this article are available in the article.
